# Positive effect of *Bifidobacterium animalis* subsp*. lactis* VHProbi YB11 in improving gastrointestinal movement of mice having constipation

**DOI:** 10.3389/fmicb.2022.1040371

**Published:** 2022-12-02

**Authors:** Hongchang Cui, Qian Wang, Congrui Feng, Chaoqun Guo, Jingyan Zhang, Xinping Bu, Zhi Duan

**Affiliations:** Qingdao Vland Biotech Group Co., Ltd., Qingdao, China

**Keywords:** probiotic, constipation, mice, YB11, *Bifidobacterium lactis*

## Abstract

**Introduction:**

The aim of this study was to investigate the effects of *Bifidobacterium animalis* subsp*. lactis* VHProbi^®^ YB11 (YB11) on attenuating sucralfate-induced constipation in BALB/c mice. The strain of YB11 exhibited favorable tolerance of simulated gastrointestinal (GI) juice. Only 0.42 Log value declined when the live cells of YB11 were co-incubated with simulated GI juice. Meanwhile, this strain also displayed perfect ability to adhere the intestinal epithelium Caco-2 cells with adhesion index of 18.5. 24 of female mice were randomized into four groups.

**Methods:**

The normal group (NOR) was fed with a normal diet, whereas the placebo group (PLA), positive group (POS), and probiotic group (PRO) were fed with sucralfate to induce constipation. After first successfully establishing the constipation model, groups NOR and PLA received the oral administration of saline solutions. Meanwhile, the POS and PRO groups were orally administered phenolphthalein and YB11 suspensions, respectively. Several indices, including fecal water content, GI transit time, short-chain fatty acids (SCFAs), intestinal neuropeptides level, and histopathology of colonic tissues, were investigated.

**Results and Discussion:**

Compared with PLA, YB11 had a positive effect in increasing the fecal water content and intestinal peristalsis. Some positive trends, including the acetic and total acids level of fecal samples, and the colonic tissue histopathology, were also observed. Furthermore, YB11 had an ability to upregulate the levels of gut excitatory neuropeptides including motilin, gastrin, and substance P, whereas it downregulated the levels of inhibitory neuropeptides including endothelin-1, somatostatin, and vasoactive intestinal peptide. We conclude that the strain YB11 has a positive impact on improving gastrointestinal mobility and reducing the severity of constipation.

## Introduction

The National Digestive Diseases Information Clearinghouse has defined constipation as “*a condition in which you may have fewer than three bowel movements a week; stools that are hard, dry, or lumpy; stools that are difficult or painful to pass; or a feeling that not all of the stools has been passed*” (Zhang, 2008). The impact of constipation on quality of life is quite significant, which indicates that constipation should not be dismissed as a trivial condition in either children or adults (Belsey et al., 2010). The pathophysiology of constipation is quite complicated; the primary causes are inherent problems of colonic sensorimotor disturbances and pelvic floor dysfunction, whereas secondary causes are associated with medications, organic disease, and systemic disease (Andrews and Storr, 2011; Girirajan et al., 2011). The approved treatments, including stool softeners, osmotic laxatives, and bulking agents, are not efficacious in all patients, only 53% of patients benefit from available therapies. The management of constipation still faces challenges due to its multifactorial causes (American College of Gastroenterology Chronic Constipation Task Force, 2005; Johanson and Kralstein, 2007; Basilisco and Coletta, 2013). Therefore, development of an easy-to-use, economical, safe, and effective strategy for the management of constipation remains a desirable goal to improve the health and well-being of patients.

The Food and Agriculture Organization of the United Nations and the World Health Organization have jointly defined probiotics as “*live microorganisms which when administrated in adequate amount, can confer a beneficial effect on the host*” (Food and Health Agricultural Organisation of the United Nations-World Health, 2002). Probiotics have been used for centuries in fermented dairy food. In the past few decades, numerous studies have demonstrated that probiotics provide a variety of health benefits to humans, especially in maintaining gastrointestinal (GI) function (Marteau et al., 2002). Several possible biological mechanisms of action are attributed to probiotics in relieving constipation conditions, including restoring the balance following intestinal dysbiosis, alternating gut sensation and modifying the function of metabolites, and regulating the intraluminal environment (Zhu et al., 2014; Zhao and Yu, 2016). For example, the short-chain fatty acids (SCFAs) and lactate acids produced by probiotics may reduce the intraluminal pH, which could shorten gut transit time by promoting colonic peristalsis, resulting in improved bowel movements (Dimidi et al., 2014). Nowadays, drugs such as metoclopramide, erythromycin, and cisapride are typically used to treat constipation, but side effects and complications have limited their application as a long-term treatment (Tsilingiri et al., 2012). On the contrary, information acquired to date shows that lactic acid bacteria have a long history of use as probiotics without any established risk to humans (Sotoudegan et al., 2019). Hence, developing new probiotic strains seems to be promising strategy for managing constipation, which can pave the way for clinical research.

The genus *Bifidobacterium* is usually associated with advantageous properties, such as treating acute diarrhea, preventing allergic disease, and alleviating constipation (Isolauri et al., 2000; Aloisio et al., 2012; Wang et al., 2017b). *Bifidobacterium animalis* subsp*. lactis* VHProbi® YB11 (referred to as YB11 hereinafter) was isolated from infant feces in our lab. This strain exhibits favorable tolerance ability on exposure to simulated gastro juice and bile salt. The mouse model of sucralfate-induced constipation is usually established to evaluate the effectiveness of therapies, as was previously reflected in relevant studies (Talley et al., 2003). In this study, we aimed to investigate the hypothesis that YB11 can attenuate the severity of constipation in a sucralfate-induced constipation model.

## Materials and methods

### Chemicals and reagents

Sucralfate (Haipu Pharmacy, China) with a concentration of 2 mg/5 ml was diluted 2-fold in 0.8% saline. Phenolphthalein (0.1 g; 100 tablets, SCR®) was dissolved in distilled water to reach a final concentration of 7 mg/ml. Gum Arabic (S11034, Yuanye®; 100 g) was dissolved into 800 ml of distilled water and boiled until the solution was transparent. Then, 50 g of activated carbon was added to the solution and boiled for 30 min. The final solution volume was adjusted to 1,000 ml after cooling.

### Strain and culture condition

YB11 was isolated from breastfeeding infant feces without probiotic product supplementation within 6 months after birth in accordance with Declaration of Helsinki and deposited at the China Center for Type Culture Collection (Wuhan, China) with accession number CCTCC M 2021905. Feed cultures were inoculated on the de-Man-Rogosa-Sharpe (MRS) agar plate medium (Hope Bio-Technology) and incubated under anaerobic conditions at 37°C for 48 h (Lee et al., 2016). The strains were activated three times before use. Fresh fermented cultures with live bacterial counts above 10^9^ CFU/ml were directly used in the animal experiments.

### Endurance in simulated Gl juice

The procedure for testing the GI survival of YB11, followed the method described by Millette with slight modification (Millette et al., 2013). The simulated gastric juice was prepared by dissolving 5.0 g of peptone (Sigma-Aldrich®), 2.5 g of yeast extract (Sigma-Aldrich®), 1 g of glucose (SCR®), and 2 g of NaCl (SCR®) in 1,000 ml distilled water and adjusted to pH 3.0 using HCl (1 N, Sigma-Aldrich®) before autoclaving. The simulated gastric juice was combined with 3.2 g of porcine mucosa pepsin (1,100 U/mg of protein, P-7000, Sigma-Aldrich®) and incubated in a water bath for 1 h at 37°C before use. The simulated intestinal fluid (SIF) was prepared by dissolving 5.0 g of peptone (Sigma-Aldrich®), 2.5 g of yeast extract (Sigma-Aldrich®), 1 g of glucose (SCR®), 6.8 g of KH_2_PO_4_, 77 ml of NaOH (0.2 N SCR®), and 3.0 g of bovine bile salt (P-8381, Sigma-Aldrich®) in 1,000 ml of distilled water and with adjustment to pH 6.8 using either HCl or NaOH before autoclaving. The SIF was combined with 1.0 g of pancreatin (P-7545, Sigma-Aldrich®) and incubated in a water bath at 37°C for 1 h before use.

The bacterial cells were harvested by centrifugation, washed thrice with 0.85% saline, and resuspended in an equal volume of phosphate-buffered saline (PBS, Aldrich®). To simulate GI transit, 1 ml of strain supernatant (10^9^ colony forming units, CFU/ml) was co-incubated with 9 ml simulated gastric juice for 2 h at 37°C. Then, 1 ml mixed solutions, taken previously, was transferred into 24 ml of SIF and incubated for 3 h at 37°C. The bacterium counts were compared before and after GI transit to evaluate the resistance of YB11 using the GB 4789.35–2016 Code (National Food Safety Standards of People’s Republic China GB 4789.35, 2016).

### Adhesion to the Caco-2 cell line

The adherence of YB11 to the Caco-2 cell line was examined as per the method previously described by Berenet, with modifications (Bernet et al., 1993). Briefly, Caco-2 monolayers prepared on a glass coverslip in a six-well plate were washed twice with PBS solution and 1 ml of cell line culture medium was then added, with a final concentration of 2 × 10^5^ cells per well. 1 ml volume of strain supernatant (5 × 10^7^ CFU/ml) was added to the walls. The suspensions were incubated in a CO_2_ chamber for 2 h at 37°C. Then, the monolayers were washed thrice with PBS, fixed with methanol (Aladdin®), and stained with Gram stain for microscopic examination. Meanwhile, the monolayers were digested with trypsin after washing by PBS to count the adherent bacteria. The adhesion ability was calculated using the following formula:

Adhesion Index (CFU/cells) = Total bacterium counts in each well/Total cell counts in each well

## Animal experiments

### Animals and experiment design

Twenty-four female specific-pathogens-free BALB/c mice aged 8 weeks were purchased from the Experimental Animals Center of Qinglongshan (Jiangsu, China). The animal permit number was SCXK (Su) 2017-0001. The mice were provided with access to standard pelleted feed and water *ad libitum* and were raised in an environment with room temperature (25 ± 2°C), 50 ± 5% relative humidity, and a 12 h light/12 h dark cycle.

The study was initiated after the mice first allowing to adaptively feed for 7 days. The mice were randomly divided (*n* = 6/group) into the normal group (NOR), placebo group (PLA), positive group (POS), and probiotic group (PRO). All of mice were fed with a regular diet from Day 1 to the end of the study. Mice from PRO group were treated with 200 μl of YB11 suspension (10^9^ CFU/ml) by gavage once daily from Day 1 to 15, whereas mice from the other three groups were treated with an equal volume of sterilized 0.8% saline. On the Day 15, Mice from PLA, POS, and PRO groups were treated with 1 ml of 50% sucralfate by gavage once daily for 2 days, followed by continued treatment with 0.5 ml of sucralfate for 3 days until the end of the study (Day 21) to induce constipation in the model (Kinds and Model, 2015). Mice from PLA, POS, and PRO groups were treated with 0.8% sterilized saline, phenolphthalein (35 mg/kg, body weight; Howarth and Sullivan, 2016) and probiotic suspensions (10^9^ CFU/ml), respectively at 1 h post-gavage. The NOR group was treated with an equal volume of sterilized saline solution during the experiments.

### Determination of fecal water content

Mice from each group were maintained in single cages after the last gavage on the Day 21, and stool samples were collected from 8:00 p.m. to 8:00 a.m. Fecal water content (%) was calculated as the difference between the dry and wet weights of stool samples according to a previously described method (Lee et al., 2012). The formula is as follow:

Water content % = (wet weight–dry weight)/wet weight * 100%

### Determination of GI transit time

The GI transit time was measured according to the method described by Verdú et al. (2009). A 200 μl volume of 3% activated carbon meal was administrated to each mouse by gavage after 24 h of the last sucralfate treatment. One hour later, the mice were euthanized by CO_2_ suffocation. The entire small intestine from the pylorus to the cecum was collected. The total length of entire small intestine and the propulsion distance of the activated carbon were measured. GI transit rate (%) = migration distance of activated carbon/the whole length of entire small intestine * 100%.

### Determination of SCFAs in feces

Mouse fecal samples were collected to determine the content of SCFAs using a gas chromatography mass spectrometer (GCMS-QP 2010 Ultra system, Shimadzu Corporation, Japan) according to the method described by Mao (Mao et al., 2016). The injection and ionization temperatures were 240 and 220°C, respectively. The carrier gas was helium (flow 2 ml /min, split ration 10:1, volume of sampling 1 μl). The massing scan was full wave. The standard curve was generated using the external standard method and the concentration of SCFAs was calculated according to the standard curve. Fecal samples (50 mg) were subjected to soaking, acidification, and extraction using saturated NaCl solution, sulfuric acid (10%), and diethyl ether, respectively. The acetic acid, propionic acid, butyric acid, and total acid contents were calculated.

### Determination of serum MTL, Gas, ET-1, SP, SS, and VIP levels

The serum neuropeptides, namely motilin (MTL), gastrin (Gas), endothelin-1 (ET-1), substance P (SP), somatostatin (SS), and vasoactive intestinal peptide (VIP) were determined using an enzyme-linked immunosorbent assay (ELISA) kit according to the manufacturer instructions (eBiosource®, United States). The standard curve was generated using the standard sample and its corresponding OD_450_ value. The blood samples collected from the mouse orbits were centrifuged at 1,650 *g* (Eppendorf®) for 15 min to obtain serum. Then, serums, biotin-labeled secondary antibody, ELISA reagents, and standard samples were added to the kits and maintained at 37°C for 60 min, then washed five times. The color A and B reagents were added and incubated at 37°C for 10 min. Finally, the termination reagent was added to obtain OD_450_ value. The concentrations of MTL, Gas, ET-1, SP, SS, and VIP were calculated.

### Histological examination

The mice were dissected to investigate the damage to colonic tissues, which were fixed in 4% neutral buffered formalin (NBF). Paraffin blocks were prepared, from which sections 5-μm in thickness were obtained. Slides were prepared, stained with hematoxylin and eosin (H&E; Promega, United States), and observed using a phase contrast microscope under 200 × magnification (LEICA ICC50, Germany; Choi et al., 2020).

### Statistical analysis

The data are presented as mean ± SD, and the differences in the mean values of each group were calculated by one-way ANOVA with Duncan’s multiple range test. *p* < 0.05 was defined as indicating statistically significant difference. Statistical analysis was performed with the software SPSS 20.0 (SPSS Inc., Chicago, IL, United States). Plots were made using GraphPad Prism 8 (GraphPad Software Inc., San Diego, CA, United States).

## Results

### Tolerance to artificial GI juice

*In vitro* assessment showed that the strain has superior survivability in simulated gastro juice and intestinal fluid. Plate counting was shown in Table 1, where the initial inoculum count was 7.49 log CFU/ml and the final surviving bacterium count merely decreased to 7.07 log CFU/ml. Our result indicated that the strain has excellent tolerance in the harsh GI environment.

**Table 1:** Effect of simulated GI juice on the viability of YB11 (log CFU/ml).

	**Initial count**	**After 2 h in simulated gastro juice**	**After 3 h in simulated intestinal fluid**
Survivability	7.49 ± 0.36	7.33 ± 0.21	7.07 ± 0.30

Values are presented in mean ± SD.

### Adherence assay in the Caco-2 cell line

**Figure 1:**
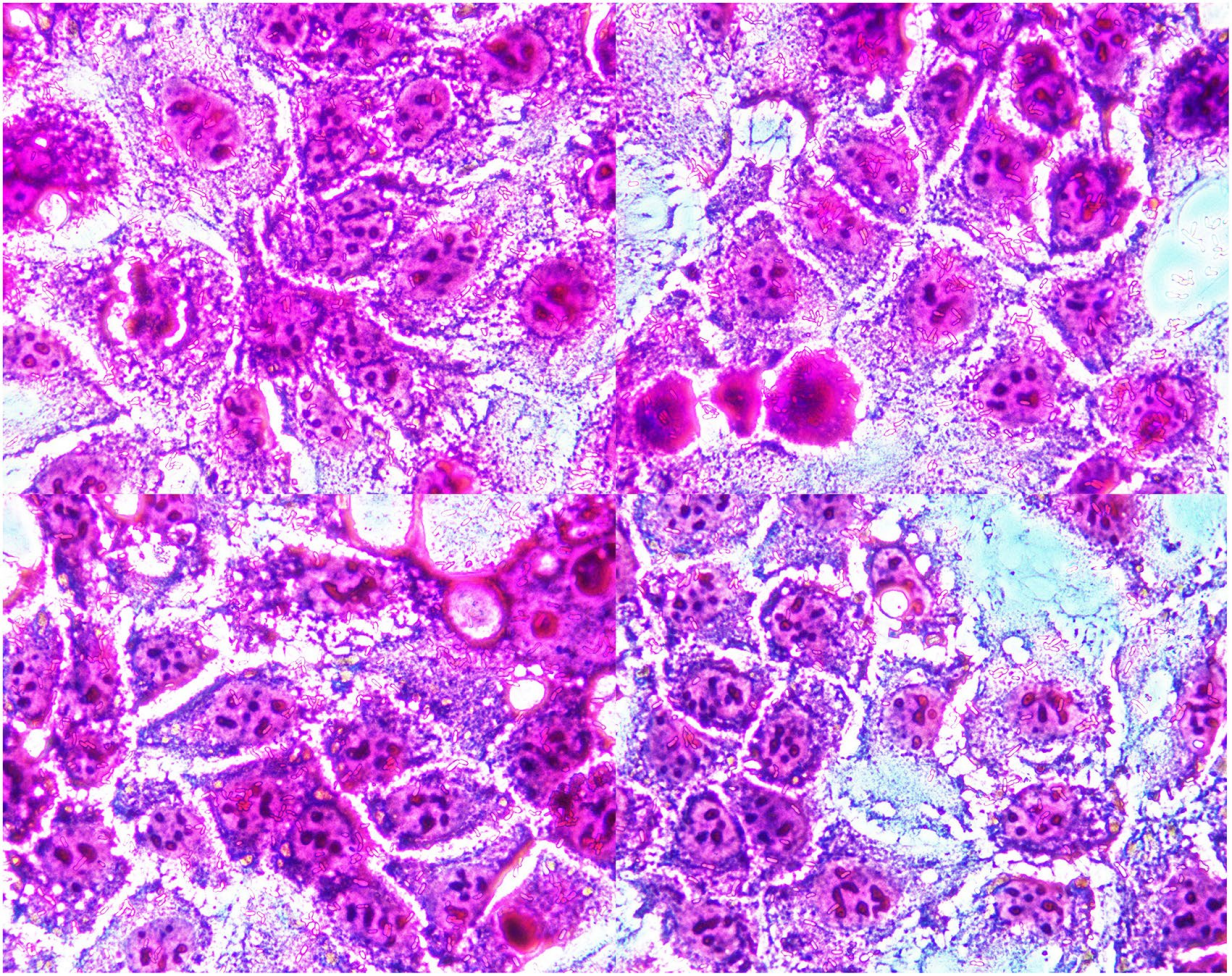
Micrographic results of adhesive ability of YB11 to Caco-2 cells.

Microscopic examination showed that bacterial clusters stuck to the surface of the polarized human intestinal epithelial Caco-2 cells (Figure 1), and the adhesion index was 18.50. Our results demonstrated that the strain of YB11 has adhesive properties toward intestinal epithelial cells.

### Fecal water contents assay

**Figure 2:**
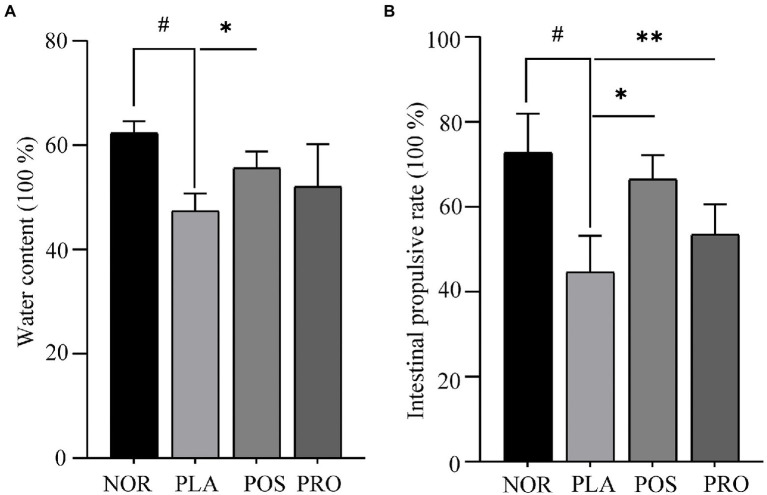
Water contents and propulsion rates of the four groups. NOR, normal group; PLA, placebo group; POS, positive group; and PRO, probiotics group. ^#^*p* < 0.05 compared with NOR; ^*^p < 0.05 compared with PLA; and ^**^p < 0.05 compared with PRO. **(A)** Fecal water count. **(B)** The activated carbon propulsion rate.

Water content is one of the principal indicators determining the improvement in constipation. The fecal sample water contents of each mouse are presented in Figure 2A. Mice from PLA, POS, and PRO groups displayed a trend of decreased fecal water content at the end of the study compared with group NOR. Mice subjected to the PLA group had significantly lower water content compared with mice belonged to the NOR group (*p* < 0.05). That observation indicated that the sucralfate-induced constipation in mouse model was successfully established. The POS group had statistically significantly higher water content than the PLA group (*p* < 0.05). Water content in PRO was higher than that in PLA, though the difference was not statistically significant.

### GI transit capability assay

**Figure 3:**
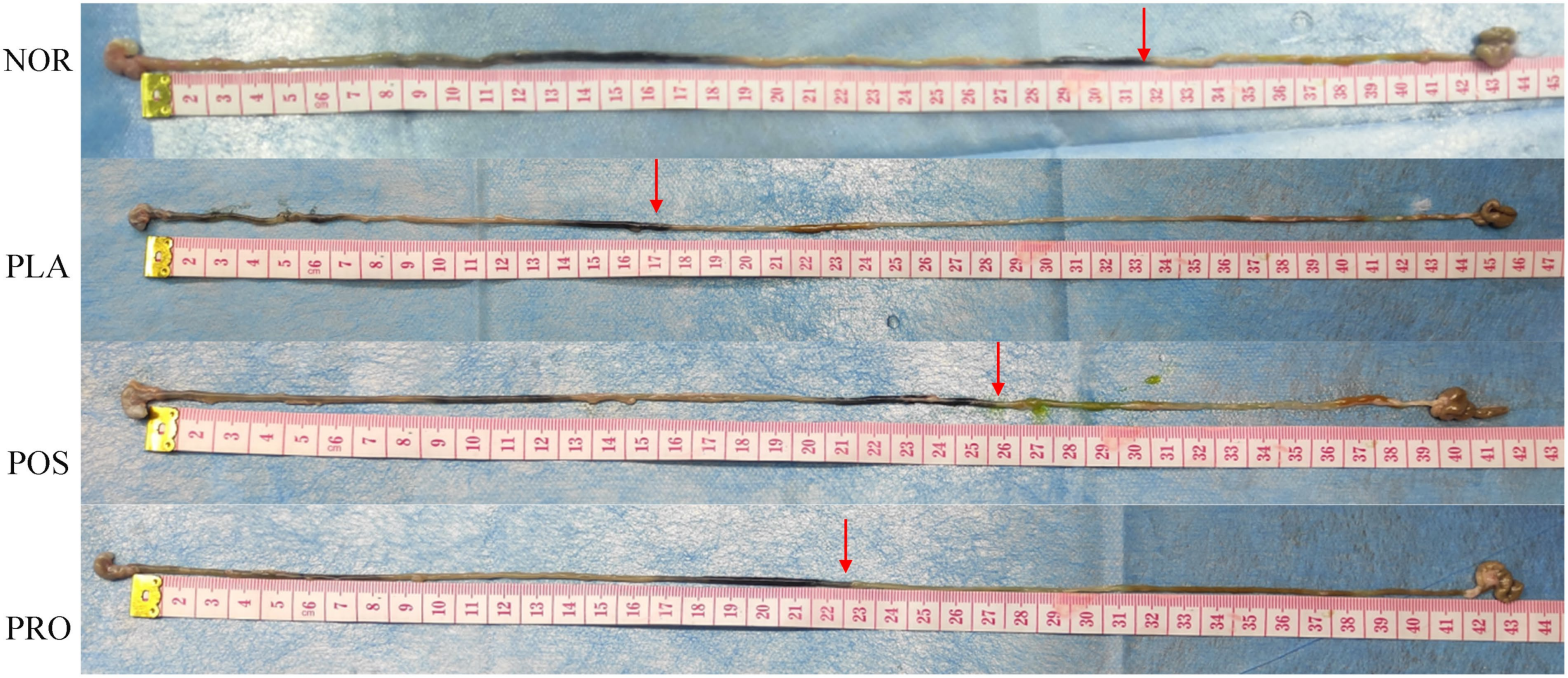
Anatomical outcomes of active carbon propulsion. NOR, normal group; PLA, placebo group; POS, positive group; and PRO, probiotics group. The red arrows indicate the peristalsis distance of activated carbon in the small intestines of mice.

The activated carbon propulsion rate is usually adopted to evaluate the intestinal peristalsis ability. The activated carbon propulsion rate and anatomical outcomes of small intestinal transit are shown in Figures 2B, 3, respectively. It was found that the activated carbon propulsion rate was significantly decreased in PLA compared with NOR (*p* < 0.05), which meant that mice in groups other than NOR suffered from constipation. The activated carbon propulsion rates in POS (*p* < 0.05) and PRO (p < 0.05) were significantly higher than that in PLA group, respectively. From the anatomical results in Figure 3, the PLA group had the lowest peristalsis distance followed by the POS, PRO, and NOR groups.

### SCFA contents in feces

The concentrations of SCFAs in feces are shown in Table 2. The concentrations of acetic acid, propionic acid, butyric acid, and total acid in NOR were maintained at a steady level. The differences in SCFA concentrations between PLA and NOR on Day 7 and 14 were not considered significant. However, the contents of acetic acid and total acids in PLA on Day 21 were significantly decreased compared with NOR (*p* < 0.001 and *p* < 0.001, respectively). The content of acetic acid on Day 21 was significantly increased in PRO compared with PLA (*p* < 0.05), whereas the butyric acid and total acid were higher than PLA though not significantly different. The acetic acid concentrations on Day 21 were significantly decreased in POS compared with PRO (*p* < 0.05), whereas no significance difference was observed comparing with PLA.

**Table 2:** Concentrations of SCFAs in feces.

Group	Time (day)	Acetic acid (mg/kg)	Butyric acid (mg/kg)	Propionic acid (mg/kg)	Total acid (mg/kg)
NOR	7	2470.00 ± 249.80	485.33 ± 81.52	576.17 ± 283.61	3531.50 ± 422.17
	14	2066.67 ± 546.50	406.50 ± 108.39^c^^d^	554.50 ± 308.85	3027.67 ± 711.86
	21	1996.67 ± 420.13^b^^c^^d^	428.00 ± 82.91	558.83 ± 289.98^d^	2983.50 ± 677.62^b^^c^^d^
PLA	7	2738.33 ± 513.36	540.50 ± 177.01	633.33 ± 221.71	3912.17 ± 690.74
	14	1796.67 ± 378.08	510.83 ± 100.89	643.50 ± 305.15	2951.00 ± 650.011
	21	963.00 ± 209.93^a^^d^	320.40 ± 170.69	372.83 ± 125.41	1656.23 ± 433.24^a^
POS	7	3281.67 ± 808.59^d^	584.33 ± 233.05	669.50 ± 282.55	4535.50 ± 1042.58
	14	2153.33 ± 464.44	612.17 ± 193.09^a^	977.67 ± 588.90	3743.17 ± 1192.22
	21	1080.00 ± 310.30^a^ ^d^	405.67 ± 148.21	406.83 ± 136.47	1892.50 ± 388.90^a^
PRO	7	2301.67 ± 1006.73^c^	543.50 ± 162.87	587.83 ± 344.71	3433.00 ± 1457.18
	14	2203.33 ± 500.55	605.67 ± 179.64^a^	893.83 ± 573.42	3702.83 ± 1156.28
	21	1541.67 ± 497.29^a^ ^b^ ^c^	323.83 ± 97.68	188.67 ± 114.60^a^	2054.17 ± 401.45^a^

NOR, normal group; PLA, placebo group; POS, positive group; and PRO, probiotic group.

a *p* < 0.05 compared with NOR.

b *p* < 0.05 compared with PLA.

c *p* < 0.05 compared with POS.

d *p* < 0.05 compared with PRO.

### Neuropeptides levels in serum

**Figure 4:**
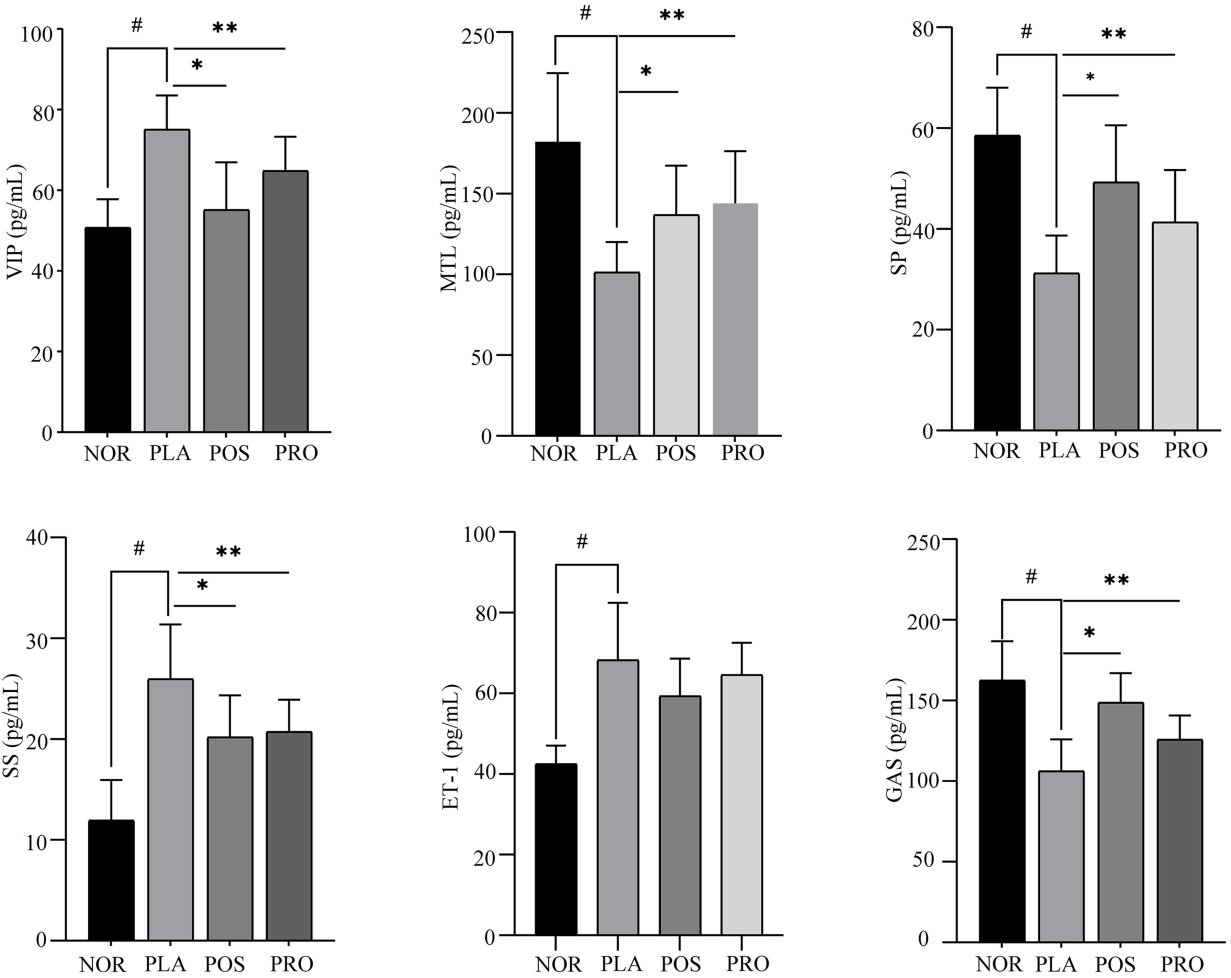
Serum levels of six neuropeptide in the four groups. NOR, normal group; PLA, placebo group; POS, positive group; PRO, probiotics group; VIP, vasoactive intestinal peptide; MTL, motilin; Gas, gastrin; ET-1, endothelin-1; SP, substance P; and SS, somatostatin. ^#^*p* < 0.05 compared with NOR; ^*^p < 0.05 compared with PLA; and ^**^p < 0.05 compared with PRO.

The effects of YB11 on constipation were also evaluated based on the measurement of VIP, MTL, SP, SS, ET-1, and Gas levels in serum. As shown in Figure 4, significant upregulation of VIP, SS, and ET-1 expression (*p* < 0.01) and downregulation of MTL, SP, and Gas expressions (*p* < 0.01) were noted in PLA compared with NOR. The expression of MTL, SP, and Gas was significantly increased (*p* < 0.01) in POS and PRO compared with PLA, whereas the expression of VIP and SS was significantly decreased (*p* < 0.01) compared with PLA. The downregulation in expression of ET-1 was not significant in POS and PRO when comparing with PLA.

### Histopathological observation of intestinal tissues

**Figure 5:**
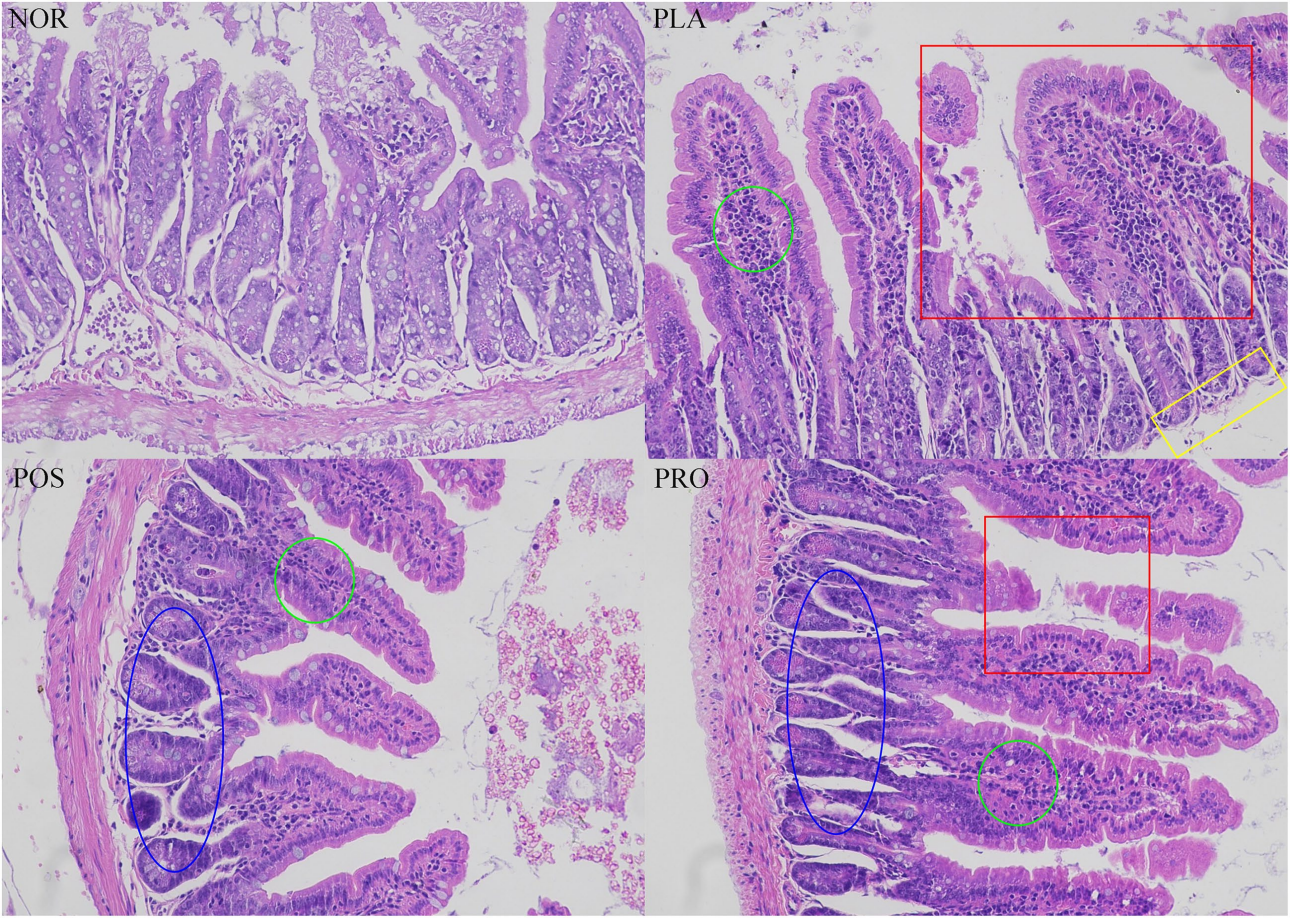
Colonic histopathology tissue of the four groups. The red square indicated the damaged intestinal villi. The yellow rectangle indicated the missing of mucosa. The green circle indicated the infiltration of inflammatory cells. The blue oval indicated the regular glands. NOR, normal group; PLA, placebo group; POS, positive group; and PRO, probiotics group.

The colonic tissue of each mouse was dissected and stained with H&E, and the loss of mucosal epithelium, the destruction of intestinal villi and goblet cells, and the infiltration of inflammatory cells were evaluated. As illustrated in Figure 5, histopathological changes were not found in NOR: the mucosa of NOR mice was basically intact, the mucosal epithelium was intact and continuous, the glands were arranged regularly and fitly, and the structure of the intestinal villi was intact. Partial congested colonic can be seen in PLA，with incomplete mucosal epithelium, shedding of intestinal villi, decreased goblet cells, and the infiltration of prevalent inflammatory cells also prevalent. In contrast, mice from POS and PRO displayed relatively integrated histological characteristics, including slightly exfoliated mucosal epithelium, a small amount of broken intestinal villi, and inflammatory cells.

## Discussion

Constipation is a common GI disorder, affecting around 9.5% of children worldwide (Koppen et al., 2018). Typical symptoms include prolonged defecation time, hard stools, and difficulty in defecation (Bharucha et al., 2013). Nonpharmaceutical treatments are usually the first step to the management of functional constipation in both children and adults, including education and lifestyle adjustments (Vriesman et al., 2020). In the past few decades, a number of studies have demonstrated that probiotics are a potential treatment modality for functional constipation. Zhao and collaborators showed that *Lactobacillus plantarum* YS-3 effectively inhibited the development of activated carbon-induced constipation in Kunming mice (Zhao et al., 2018). Wang et al. confirmed that *Bifidobacterium* spp. displayed specific-species effects in relieving the symptoms of constipation in BALB/c mice by increasing the production of acetic acid and regulating the relative abundance of *Alistipes*, *Lactobacillus*, *Clostridium*, and *Odoribacter* (Wang et al., 2017a,b). Moreover, a plentiful of random controlled trials have studied the efficacy of probiotics in the treatment of constipation (Tabbers et al., 2011; Madempudi et al., 2020; Shang et al., 2022). For instance, the administration of *B. bifidum* CCFM16 efficaciously ameliorated the severity of constipation in Chinese adults by regulating SCFA metabolism (Wang et al., 2022).

The ability of probiotics to tolerate gastro juice and intestinal fluids is the fundamental requirement for colonizing the GI, thereby achieving its greatest effects on target sites. The pH values of the stomach and small intestine have been estimated to be around 3 and 6.8, respectively (Evans et al., 1988). Our study revealed that the viable cells of YB11 only decreased by 0.42 Log units after 5 h of incubation, which means that this strain can survive in the hostile GI environment. Having the characteristic of adhesion to the intestinal epithelial cells is another important prerequisite for colonization. This is regarded as a possible protective mechanism of action for intestinal epithelial cells against pathogens through providing competition for binding sites and nutrients or modulation of immune system (Westerdahl et al., 1991; Schiffrin et al., 1997; Salminen et al., 1998). The Caco-2 cell line originally isolated from the human colon adenocarcinoma is frequently used as an *ex vivo* model of the intestinal epithelium to study bacterial adhesion properties (Fogh et al., 1977). Regarding the assessment of adhesion, the results obtained from microscopic examination are in agreement with those of cell enumeration measurements, since a high number of bacteria were observed to adhere to the cell surface. Zuo et al. investigated the ability of 13 *Bifidobacterium*. spp. strains plus *B. animalis* subsp*. animalis* Bb-12 to adhere to the Caco-2 cell line. The adhesion index values of these bacteria varied from 2.83 to 180.09, and that of *B. animalis* subsp*. animalis* Bb-12 was 30.38 (Zuo et al., 2016). Our results were consistent with those of previous research, which means that YB11 has a relatively high adhesive ability when considering the intestinal epithelial cells. We could, therefore, hypothesize that this strain may colonize the intestinal tract by comparing the survival rate in simulated gastric and intestinal fluid and adhesive ability of YB11 to enteric epithelial cells.

A constipation mouse model was established by oral administration of sucralfate, which is a gastric mucosa protectant that is mainly used to prevent and treat GI mucosal injury in clinical practice (Bonassa et al., 2015). The mechanism leading to constipation involves in inhibiting the intestinal peristalsis and increasing the water absorption in the intestine (Andrews and Storr, 2011; Kudaravalli and John, 2021). Fecal water content is an important indicator of fecal status, as prolonged retention of feces in the intestinal tract can lead to excessive water absorption. Wang et al. found that the administration of *B. adolescentis* improved the fecal water content in loperamide-induced mice (Wang et al., 2017b). The movement frequency of intestinal content can be measured by the intestine propulsion rate and this can be observed by the activated carbon propelling rates. The higher the activated carbon propelling rates, the lower the degree of constipation in mice. In our study, mice which received sucralfate displayed apparent constipation symptoms, including significantly decreased fecal water content and a small intestinal propulsion rate compared with normal mice. These symptoms were relieved in the POS and PRO groups, indicating that the YB11 may relieve constipation by promoting the fecal water content and peristalsis. In addition, we also found decreased damage to intestinal villi in POS and PRO groups, which mean that mice in these groups had improved peristalsis ability.

It has been reported that SCFAs are the end-products of bacterial fermentation of polysaccharides in the mammalian colon, and they can reduce the intestinal pH and thereby inhibit the proliferation of pathogens and stimulate peristalsis (Kamath et al., 1988). Acetic, propionic, and butyric acids are three of most abundant SCFAs in the feces and are the major SCFAs involved in mammalian physiology (Li et al., 2015). Chen et al. demonstrated that *Lactobacillus paracasei* NTU 101 significantly improved the SCFAs levels in loperamide-induced constipation rats compared with control groups (Chen et al., 2020). The intestinal osmotic pressure and the water content of intestinal contents will increase as the content of acetic acid increases, followed by stimulating the intestinal wall and increasing the intestinal peristalsis (Gupta et al., 2015). Mice from PRO group had an increased concentration of acetic acid, thus leading to speculate that YB11 may attenuate the severity by producing acetic acid.

Mice with sucralfate-induced constipation exhibited damaged intestinal villi and decreased mucosal thickness. The histopathology results supported assertation that YB11 could attenuate damage to colonic tissue compared with the untreated constipation groups. Slow intestinal peristalsis and fecal induration caused by constipation are mainly related with the integrity of the colonic tissue. Wang et al. argued that *B. adolescentis* CCFM 667 could produce SCFAs from carbohydrates that the small intestine could not absorb but could potentially promote the colonic peristalsis (Wang et al., 2017b). Li et al. found that *Lactobacillus plantarum* NCU116 promotes the production of SCFAs, leading to decreased mucosal damage in loperamide-induced mice (Li et al., 2015). SCFAs exert effects in the regulation of proliferation and differentiation of intestinal epithelium cells through binding to cell surface receptors (Astakhova et al., 2016). Therefore, it appears that the strain YB11 could ameliorate colonic tissue damage by regulating the production of SCFAs, particularly the marked upregulation of acetic acid.

More importantly, we also found that YB11 can regulate the contents of neuropeptides that are related to peristalsis. MTL, Gas, and SP are regarded as excitatory gastrointestinal neuropeptides that can stimulate the gastrointestinal peristalsis, smooth muscle contractions, and the secretion of gastric acid and pepsinogen (Chen et al., 2012; Reigstad et al., 2015; Lee et al., 2019), whereas ET-1, SS, and VIP are the inhibitory neuropeptide that can inhibit the release of gastrointestinal hormone, excitation of cholinergic nerves, and in addition to stimulating the relaxation of sphincters (Xu et al., 2012; Jiang et al., 2020). Zhao et al. demonstrated that *Lactobacillus plantarum* YS2 exhibits an activity to attenuate activated carbon-induced constipation in Kunming mice through upregulating the serum levels of MTL and SP (Zhao et al., 2019). A previous study reported that the administration of *L. plantarum* CQPC05 significantly increased the serum Gas and decreased the SS compared with the control group. In the present study, we found that MTL, Gas, and SP levels were significantly higher in PRO than that in PLA, while SS, ET-1, and VIP levels were lower. This indicated that the strain YB11 may attenuate the severity of constipation by regulating neuropeptides levels.

Our present study also has several deficiencies. A positive effect of YB11 on attenuating sucralfate-induced constipation in mice was observed. However, the mechanism of actions of YB11 in ameliorating constipation need to be further investigated. For example, whether alternation of microbiota compositions involved in the regulations of related indices is yet to be determined. Therefore, illustrating the gut flora changes is a pivotal step to clarifying the probiotic mechanism of YB11. In addition, it is essential to enhance the level of supporting evidence in regard to YB11 by implementing randomize, double-blind, and placebo control trials.

In summary, our results clearly show that YB11 could proliferate in the harsh gut environment, increase the fecal water content, and stimulate the intestinal peristalsis function. In addition, it can increase the serum levels of MTL, Gas, and SP and decrease the levels of ET-1, SS, and VIP. Furthermore, YB11 regulates the SCFAs levels, thus improving the integrity of colonic tissues. In the future, YB11 could be used as live therapeutics for the treatment of chronic constipation.

## Data availability statement

The raw data supporting the conclusions of this article will be made available by the authors, without undue reservation.

## Ethics statement

The animal study was reviewed and approved by Nanjing University of Chinese Medicine.

## Author contributions

All authors listed have made a substantial, direct, and intellectual contribution to the work and approved it for publication.

## Funding

This work was supported by the Mountain Tai New Strategy Industry Leader Program (Grant No. tscy20180317). This study was also received funding from Qingdao Vland Biotech Group CO., Ltd. This company was not involved in the study design, collection, analysis, data interpretation, the writing of this article, or the submission to it for publication.

## Conflict of interest

HC, QW, CF, CG, JZ, XB, and ZD were employed by Qingdao Vland Biotech Co., Ltd.

## Publisher’s note

All claims expressed in this article are solely those of the authors and do not necessarily represent those of their affiliated organizations, or those of the publisher, the editors and the reviewers. Any product that may be evaluated in this article, or claim that may be made by its manufacturer, is not guaranteed or endorsed by the publisher.
